# Supplementary material for: A novel nomogram based on the patient’s clinical data and CT signs to predict poor outcomes in AIS patients
Source: PeerJ. 2025 Jan 6;13:e18662. doi: 10.7717/peerj.18662 (PMC11716008; doi:10.7717/peerj.18662)
Supplement: Supplemental Information 4 [file peerj-13-18662-s004.doc]

Label: Label=outcome 0=good outcome；1=poor outcome;

Gender: 0=female 1=male;

dd: dd=insular ribbon sign 0= no 1=yes;

ASPECT ≥ 6: 0 = ASPECT ≥ 6；1= ASPECT ＜6

Location of lesion: 0=right hemisphere ;1=left hemisphere;

Thrombolysis、Hypertension、Previous stroke、Diabetes、Atrial fibrillation、IAC、lacunar infarction、brain tissue swelling sign、lenticular nucleus obscuration、encephalomalacia、HMCAS、Coronary disease、BGC、Leukoaraiosi、Brain atrophy：0 = no ( No such CT signs/medical history); 1=yes ( Have such CT signs/medical history);

The remaining variables are continuous variables.
