# Supplementary material for: A novel nomogram based on the patient’s clinical data and CT signs to predict poor outcomes in AIS patients
Source: PeerJ. 2025 Jan 6;13:e18662. doi: 10.7717/peerj.18662 (PMC11716008; doi:10.7717/peerj.18662)
Supplement: Supplemental Information 5 [file peerj-13-18662-s005.docx]

Table 1

|  | Overall N=161 | Training N=113 | Validation N=48 | P-value |
| --- | --- | --- | --- | --- |
| **Demographic data, n (%)** |  |  |  |  |
| Gender, male (%) | 117 (72.7) | 81 (71.7) | 36 (75.0) | 0.811 |
| Age, years (mean (SD)) | 63.12 (12.08) | 63.33 (12.25) | 62.65 (11.77) | 0.744 |
| **Vascular risk factors, n (%)** |  |  |  |  |
| Hypertension, yes (%) | 75 (46.6) | 55 (48.7) | 20 (41.7) | 0.521 |
| Diabetes, yes (%) | 31 (19.3) | 24 (21.2) | 7 (14.6) | 0.447 |
| Atrial fibrillation, yes (%) | 20 (12.4) | 15 (13.3) | 5 (10.4) | 0.809 |
| Previous stroke, yes (%) | 26 (16.1) | 19 (16.8) | 7 (14.6) | 0.906 |
| Coronary disease, yes (%) | 18 (11.2) | 10 ( 8.8) | 8 (16.7) | 0.243 |
| **Imaging findings, n (%)** |  |  |  |  |
| HMCAS, yes (%) | 25 (15.5) | 19 (16.8) | 6 (12.5) | 0.650 |
| BGC, yes (%) | 33 (20.5) | 26 (23.0) | 7 (14.6) | 0.318 |
| Leukoaraiosis, yes (%) | 54 (33.5) | 39 (34.5) | 15 (31.2) | 0.827 |
| Brain atrophy, yes (%) | 73 (45.3) | 48 (42.5) | 25 (52.1) | 0.344 |
| IAC, yes (%) | 28 (17.4) | 22 (19.5) | 6 (12.5) | 0.401 |
| encephalomalacia, yes(%) | 28 (17.4) | 20 (17.7) | 8 (16.7) | >0.999 |
| lacunar infarction, yes(%) | 91 (56.5) | 63 (55.8) | 28 (58.3) | 0.898 |
| lenticular nucleus obscuration, yes(%) | 12 ( 7.5) | 6 ( 5.3) | 6 (12.5) | 0.207 |
| insular ribbon sign, yes(%) | 11 ( 6.8) | 8 ( 7.1) | 3 ( 6.2) | >0.999 |
| brain tissue swelling sign, yes(%) | 14 ( 8.7) | 9 ( 8.0) | 5 (10.4) | 0.842 |
| **Baseline data** |  |  |  |  |
| Location of lesion，right (%) | 78 (48.4) | 20 (41.7) | 58 (51.3) | 0.342 |
| ASPECT≥6, yes (%) | 40 (24.8) | 28 (24.8) | 12 (25.0) | >0.999 |
| ASPECT (median [IQR]) | 9.00 [8.00, 10.00] | 9.00 [7.50, 10.00] | 9.00 [8.00, 10.00] | 0.902 |
| NIHSS score (median [IQR]) | 15.00 [9.00, 21.00] | 14.00 [9.00, 21.00] | 16.00 [10.00, 21.25] | 0.540 |
| SBP, mmHg (mean (SD)) | 142.89 (24.58) | 141.59 (23.69) | 145.96 (26.56) | 0.304 |
| DBP, mmHg (mean (SD)) | 84.87 (14.46) | 84.16 (12.82) | 86.54 (17.79) | 0.341 |
| IV thrombolysis, yes (%) | 78 (48.4) | 60 (53.1) | 18 (37.5) | 0.101 |
| **Laboratory data** |  |  |  |  |
| NA, mmol/L (median [IQR]) | 140.60 [138.00, 143.00] | 140.00 [138.00, 142.90] | 141.70 [138.23, 143.07] | 0.406 |
| K, mmol/L (median [IQR]) | 3.89 [3.60, 4.10] | 3.90 [3.60, 4.14] | 3.88 [3.60, 4.07] | 0.478 |
| Ca, mmol/L (median [IQR]) | 2.26 [2.15, 2.38] | 2.24 [2.15, 2.38] | 2.27 [2.17, 2.38] | 0.596 |
| Cl, mmol/L (median [IQR]) | 103.00 [101.00, 105.00] | 103.00 [101.00, 105.00] | 103.00 [100.75, 104.78] | 0.897 |
| Crea, umol/L (median [IQR]) | 70.22 [61.20, 92.54] | 69.71 [61.20, 93.08] | 70.34 [61.02, 85.82] | 0.515 |
| LDH, U/L (median [IQR]) | 220.00 [187.00, 263.00] | 222.00 [189.00, 264.00] | 218.00 [176.75, 256.50] | 0.325 |
| LDH1, U/L (median [IQR]) | 30.00 [24.00, 37.00] | 30.00 [24.00, 38.00] | 30.00 [23.75, 36.25] | 0.826 |
| HGB, g/L (median [IQR]) | 141.00 [126.00, 152.00] | 140.00 [126.00, 151.00] | 142.00 [126.00, 155.00] | 0.645 |
| WBC, ×10^9/L (median [IQR]) | 9.30 [7.37, 11.33] | 9.51 [7.76, 11.38] | 9.02 [7.08, 10.78] | 0.228 |
| RBC, ×10^12/L (median [IQR]) | 4.67 [4.30, 5.11] | 4.68 [4.32, 5.11] | 4.65 [4.24, 5.04] | 0.429 |
| Urea, mmol/L (median [IQR]) | 5.61 [4.63, 6.82] | 5.62 [4.74, 6.84] | 5.46 [4.34, 6.73] | 0.665 |
| Glu, mmol/L (median [IQR]) | 6.68 [5.86, 8.87] | 6.77 [5.98, 8.83] | 6.57 [5.80, 9.70] | 0.720 |
| Plt, ×10^9/L (median [IQR]) | 232.00 [186.00, 267.00] | 239.50 [200.25, 263.25] | 226.00 [181.00, 270.00] | 0.439 |
